# TCP-WBQ: a backlog-queue-based congestion control mechanism for heterogeneous wireless networks

**DOI:** 10.1038/s41598-022-07276-3

**Published:** 2022-03-01

**Authors:** Junyong Tang, Yufei Jiang, Xirong Dai, Xiangyang Liang, Yanfang Fu

**Affiliations:** Department of Computer Science and Engineering, Xi’an Technological University, Xi’an, 710021 China

**Keywords:** Computer science, Information technology

## Abstract

In heterogeneous wireless networks, random packet loss and high latency lead to conventional TCP variants performing unsatisfactorily in the case of competing communications. Especially on high-latency wireless links, conventional TCP variants are unable to estimate congestion degrees accurately for fine-grained congestion control because of the effects of random packet loss and delay oscillations. This paper proposes a TCP variant at the sender side to identify congestion degrees, namely TCP-WBQ, which quickly responses to the real congestion and effectively shields against random packet loss and oscillations of latency time. The proposed algorithm of congestion control firstly constructs a backlog-queue model based on the dynamics of the congestion window, and deduces the two bounds of the model which delimit oscillations of the backlog queue for non-congestion and random packet loss respectively. TCP-WBQ detects congestion degrees more accurately and thus implements the corresponding schemes of adjusting the congestion window, maintaining a tradeoff between high throughputs and congestion avoidance. The comprehensive simulations show that TCP-WBQ works efficiently in bandwidth utilization with single and multiple bottleneck scenarios, and achieves high performance and competitive fairness in heterogeneous wireless networks.

## Introduction

The increasing interest in heterogeneous networks has heightened the need for maintaining high throughput in wireless communications^1–3^. In general, heterogeneous networks include diverse links with different communication features^4^, e.g., the unreliable satellite channel, the reliable and high-rate optical fiber opposite to the former, and the high-latency wireless link that is vulnerable to noise interference. These asymmetrical and diverse links disadvantage wireless communications. Noise interference and the high latency of the wireless link lower the throughput of wireless connections and prevent the wireless stream from efficiently occupying the shared capacity of a bottleneck link. The reason is that (1) different data streams simultaneously travel across these asymmetrical links such that continuous packets in the same wireless stream disperse in the buffer of the intermediate router due to the high latency of the wireless link^5^, which makes the TCP receiver spend more time to deal with the packet disorder and further slows the transmission rate down. The increased delay even induces, in extreme cases, the retransmission timeout (RTO) event of Transmission Congestion Protocol (TCP). (2) In addition, compared with robust wired links, the wireless link randomly drops more packets in the condition of noise interference. While the random packet loss is not derived from communication congestion, some TCP mechanisms falsely regard it as a congestion signal and, therefore, reduce the transmission rate to avoid this spurious congestion, which negatively impacts the utilization of the shared capacity.

The focus of resolving the above challenges has been to design the appropriate TCP variant to improve throughput and performance of wireless communications in heterogeneous networks. Generally, conventional TCP variants are classified into two categories, i.e., loss-based and delay-based TCP variants^6^, according to the recognition of congestion. Corresponding to the first category, TCP-NewReno^7^ and Hybla^8,9^ treat the case of TCP source receiving the fixed triple duplicate acknowledgments (Acks) as congestion occurring. Although the loss-based TCP variants can quickly implement congestion avoidance to respond to the congestion signal on wireless channels^10^, they lack the ability to identify whether the signal indicates random packet loss. The indiscriminate and frequent congestion avoidance enables the wireless stream to underutilize the shared capacity^11–13^.

Compared with the loss-based TCP, the delay-based variants, such as Vegas^14^ and DC-Vegas^15^, are less sensitive to the random packet loss^16^ because they mainly measure the round trip time (*Rtt*) of transmitted packets to estimate whether congestion occurs or not. The delay-based variants perform well in high-speed and long-distance networks^17^ but poorly in competitive wireless communications. In the competitive case, the packets belonging to the same wireless stream are irregularly scattered to the bottleneck link, causing these packet Acks in bursts and oscillations. Therefore, accurately sampling *Rtt* becomes difficult, that is, estimating congestion is also uncertain. For the above reason, the delay-based variants incline to estimate congestion seriously and thus lose competitiveness in the heterogeneous wireless network. In addition, a few hybrid solutions, such as TCP-W^18^ and TCP-W + ^19^, have been proposed to take advantage of the loss-based and delay-based TCP variants. These hybrid solutions are very promising for utterly wireless networks, but for heterogeneous wireless networks, they are not suitable.

Summarily, in asymmetrical communications, current TCP variants on wireless links either use the same congestion signal undistinguishing the congestion packet loss and the random packet loss, or are difficult to estimate congestion accurately due to the high wireless delay.

In this paper, we extend TCP-W and therefore propose a high-performance and high-fairness TCP mechanism or variant, namely TCP-WBQ, for heterogeneous wireless networks. TCP-WBQ was inspired by the twofold.

- The analysis of dynamic queue limits (DQL)^20^ and the queue size difference^21^ in intermediary routers revealed the appropriate packet queue not only keeps transmission latency stable but effectively controls congestion for TCP streams.
- In TCP-W and loss-based TCP variants, signals of the fixed triple duplicate Acks and the RTO event can indicate congestion as quickly as possible, the congestion recognition that is important to the high-latency wireless link.

Based on the above insight, we design the TCP-WBQ which combines the rapid response of TCP-W to congestion and the steady latency of the packet queue. In detail, we first model the backlog queue based on behavior of the TCP sender transmitting packets. That the backlog queue is created is similar to the way of the packet queue in the intermediate router’s buffer, but it locates only at the TCP source end and does not require Explicit Congestion Notification (ECN) support or network device modifications. Subsequently, we analyze the backlog queue dynamics by considering non-congestion, and spurious congestion caused by the random packet loss, and therefor conclude two bounds of the backlog queue accordingly. The bounds quantify three congestion degrees in terms of the range of the backlog queue. TCP-WBQ reduces the congestion window (*cwnd*) adaptively in the light of the congestion degree while receiving the triple duplicate Acks or RTO signal, and adopts the multiplicative increase in *cwnd* instead of the traditional additive increase. Either the adaptive decrease or the multiplicative increase aims to avoid the true congestion to achieve high throughput on the wireless link. Experiments show that TCP-WBQ (1) efficiently improves wireless throughput; (2) significantly avoids the true congestion; and (3) maintains graceful fairness to the wireless/wired streams.

The remainder of this paper is organized as follows.  “Related work” Section discusses the related work in TCP variants improving performance in heterogeneous networks. Next, in Backlog queue” Section, the backlog queue mode is introduced, and its advantage for identifying the congestion degree is illustrated by two simulations. Subsequently, “TCP-WBQ mechanism” Section describes the mechanism details of TCP-WBQ and presents some analysis. Experimental results are provided in “Evaluation” Section. Finally, we conclude the paper in “Conclusion” Section.

## Related work

Recently, many TCP variants for congestion control are proposed for wireless networks. We summarize the existing work promoting the performance of wireless streams.

A-CAFDSP^22^ respectively considers the carrying capacity, the dissimilar characteristics, and the background traffic intensity of parallel links to select the efficient links for concurrent transmissions. Early window tailoring (EWT), a new network-return technique solution, was proposed in^23^. By scaling the TCP receiver’s *cwnd* in accord with the gateway’s available memory space, EWT maintains a satisfied throughput required by specific applications within a given packet-loss rate. TCP-NACK^24^ inserts a negative acknowledgment (NACK) flag into the TCP segment to retransmit only the lost packet without specially reducing the transmission rate. The solution mainly considers that unacknowledged packets in the receiver’s buffer affect the sender’s *cwnd*. Meanwhile, TCP-NACK establishes a Markov state space in the congestion avoidance phase to predict the error probability of each packet so as to increase *cwnd* efficiently to achieve the high utilization of link capacity. It is at the expense of extending *Rtt*, thus degrading the performance of wireless networks. However, these solutions are deployed at not only the TCP sender but also intermediate devices, and thus the deployment cost and complexity are high.

TCP-W + , inherited from TCP-W, shields the oscillation of *Rtt*^25^ by continuously detecting the Acks’ arrival rate, and thus estimates the occupied bandwidth. TCP-W + reduces *cwnd* and the start threshold (*ssthresh*) according to the estimated bandwidth (*EB*) rather than recklessly cutting these values^26^ as loss-based variants do. Nevertheless, due to adopting additive increase in *cwnd*, TCP-W + , in the multi-traffic competition, occupies the bandwidth of the bottleneck link slower than the loss-based TCP variants over better-wired links^27^. This unfairness increases the latency upon the wireless link, thus enabling TCP-W + to often under-estimate the available bandwidth.

In^28^, the scheme considers the discrimination of packet loss based on machine learning in wireless networks. This scheme learns how to distinguish the true congestion from packet loss by the multi-layer perceptron. According to the learning results, the congestion control classifies the reasons for packet error. It performs well in the case of low bandwidth utilization. However, with the utilization increasing, both noise and congestion events increase the latency, the high latency that leads to inaccurate decision-making.

To adaptively adjust rather than linearly increase *cwnd*, TCP-ACC^29^ proposed a real-time reordering metric to infer the probabilities of packet loss and RTO events. The mechanism measures and estimates *Rtt* according to the inferred probabilities. During the congestion avoidance, it transmits as many packets as possible by setting appropriate *cwnd* based on the probabilities. Although its *cwnd* setting addresses the problems of packet loss and reordering in wireless networks, it is not suitable for the competition of multiple flows.

Hybla stemmed from TCP-NewNeno is increasingly recognized for yielding high throughput, especially over the satellite channel. Due to *cwnd* exponentially growing, Hybla is superior to TCP-NewReno in terms of throughput on wireless connections with longer *Rtt*. Nevertheless, when the *Rtt* and packet loss exceed a certain range, its performance drops dramatically because the *cwnd* threshold in the slow start phase is very low.

Although the above-related TCP variants achieved very important contributions for wireless communications, they either are difficult to be deployed, or lack overall consideration of the random packet loss and high latency of the wireless link. These solutions in the heterogeneous wireless network still have low throughput when the wireless stream they controlled compete with other wired streams.

## Backlog queue

### Modeling

To describe the backlog queue and its buildup, we firstly approximate the actual bandwidth by the estimated bandwidth (*EB*) used in TCP-W/TCP-W + . The *EB* can be denoted in the continuous time form

1 $$EB \cong \mathop {\lim }\limits_{\Delta t \to 0} \frac{d(t + \Delta t) - d(t)}{{\Delta t}} = \frac{\partial d(t)}{{\partial t}}$$

Since *d*(*t*) is the acknowledged bytes that a TCP receiver confirms receipt of bytes from a TCP sender at time *t*, the actual output in packets, *c*_*act*_(*t*), is denoted as

2 $$c_{act} (t) = EB/MSS$$

Note that *MSS* is the maximum size of one TCP segment. Supposed all segments’ MSS in TCP layer is equal for convenient description, and *c*_*act*_ can be regarded as the number of transmitted packets at the TCP sender during one *Rtt*. The backlog queue is considered as the count of packets waiting for being transmitted in the TCP sender’s buffer, its model is defined as

3 $$q(t) = \left\{ {\begin{array}{*{20}l} {w(t) - c_{act} (t)Rtt(t),} & {if\;w(t) > c_{act} (t)Rtt(t)} \\ {0,} & {if\;w(t) \le c_{act} (t)Rtt(t)} \\ \end{array} } \right.$$

where *w*(*t*) denotes the value of *cwnd*, which is the number of packets to be sent at time *t*. From (1–3), it is seen that the backlog queue considers the effect of *Rtt* and transmitted packets. Since congestion decreases the transmission rate^30^ of a TCP sender, the decrease interferes with more packets not being sent, and thus these detained packets form a backlog queue.

**Figure 1:**
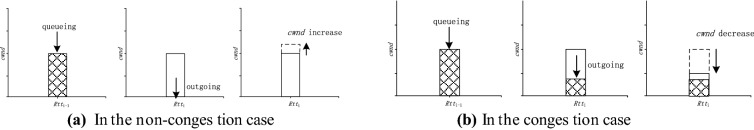
Evolution of the backlog queue.

We have drawn two schematic diagrams to show the process of *cwnd* increase and the backlog queue buildup, respectively. As shown in Fig. 1a, in the case of none-congestion, the packets to be transmitted queue up at the time of the previous round (*Rtt*_i−1_). After transmitting all packets of *cwnd*, i.e., outgoing process, the sender keeps *cwnd* unchanged until one round time (*Rtt*_i_). When the sender receives all Acks, the congestion mechanism regards the case as non-congestion and thus increases *cwnd*. Generally, for AIMD (Additive Increase and Multiplicative Decrease) congestion mechanism, the increment is one. Figure 1b shows how the backlog queue cumulates when congestion occurs. At *Rtt*_i−1_, the queuing process in the case of congestion is the same as under non-congestion, but the sender can receive congestion signals at *Rtt*_i_. These signals mean at least one packet is lost, which requires the sender retransmits the missing packets. Thus, these retransmitted packets sojourn in *cwnd*. Therefore, TCP cuts *cwnd* down to avoid congestion and retransmits the lost packets. The retransmission blocks subsequent packets to be sent, the blocked packets that thus form the backlog queue.

The backlog queue locates at the TCP sender even though its formation is similarly to the packet queue in the intermediary router. Designing a TCP variant based on the backlog queue completely follows the sender-side-only criteria for easy deployment and low cost.

### Motivation for TCP-WBQ

The congestion recognition of TCP-WBQ was inspired by the active queue management (AQM). We supposed that the size of the backlog queue can reflect the fine-grained congestion degrees as AQM algorithms do. If the supposition is true, the backlog queue can overcome the drawback of TCP-W which roughly detects congestion.

To introduce the advantage of the backlog queue recognizing congestion, we first illustrate the process of the congestion detection in TCP-W. The detection includes three steps. The first step, receiving congestion signals: TCP-W uses the packet loss (triple duplicate Acks) and RTO as congestion signals like loss-based TCP variants. Since the congestion signals are driven by the corresponding events, they are sensitive to congestion events. The second step, estimating bandwidth: when congestion signals arrive at the sender, TCP-W samples *Rtts* and thus estimates the real-time bandwidth according to (1). The third step, setting parameters: according the bandwidth estimation, TCP-W adjusts *cwnd* and *ssthresh*. The two parameters’ setting in TCP-W is shown in Table 1.

**Table 1:** TCP-W setting.

Congestion signals	*ssthresh*	*cwnd*
Packet loss (Triple duplicate Acks)	*B(t)*∙*Rtt*_min_/MSS	*ssthresh*
RTO	Max(*(B(t)*∙*Rtt*_min_)/MSS, 2)	1

As mentioned above, TCP-W leverages *EB* reflecting the congestion degree to adjust *cwnd*. However, it is difficult to acquire the accurate degree because of the frequent random packet loss and high latency on the wireless link.

To overcome the drawback, we now implement two experiments to show the superiority of the backlog queue detecting congestion. The tests mimic the competitive communications with 50 senders sending wireless streams to the same receiver. The different delay times on the wireless links are set, including 2.5 ms, 25 ms, 50 ms, 100 ms, and 150 ms, to aggravate the congestion degree gradually. Meanwhile, some random reverse UDP flows are selectively introduced into wireless links as background traffic. These streams are driven by TCP-W. In both tests, the senders transmit packets at a fixed rate (10 Mbps) during the same time (30 s).

**Figure 2:**
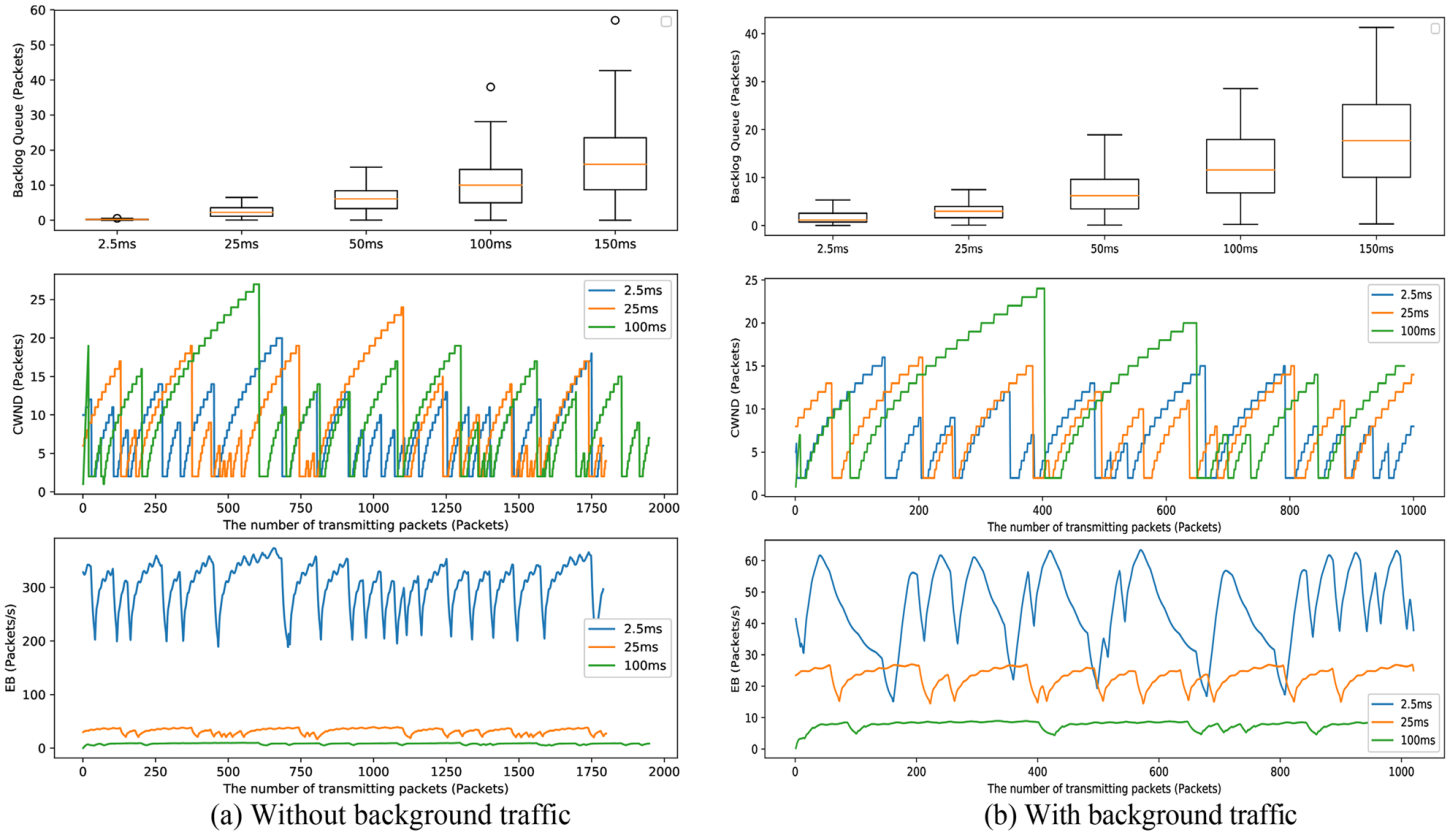
Variations of the backlog queue, *cwnd*, and *EB* for different delay times.

Figure 2a shows the average backlog queues in different delay times grow with the increase of the congestion degree in the absence of background traffic. However, the change of *cwnd* is not significant with the delay time increasing. Similar to the variation in *cwnd*, EB is superior only in the delay of 2.5 ms, but the differences of EB are not distinct in the cases of the delay time greater than 2.5 ms. Figure 2b shows the similar variations of the backlog queue size, *cwnd* and EB for different congestion degrees in the presence of background traffic. The two tests demonstrate the size of the backlog queue is positively related to congestion degrees. Reversely, the *cwnd* and *EB* fluctuate seriously and are unable to show the congestion degree clearly. Thus, Fig. 2 only shows the comparisons of *cwnd* and EB for the delay of 2.5 ms, 25 ms and 100 ms for conciseness.

Accordingly, replacing the unsteady *cwnd* and *EB* with the backlog queue contributes to reflect the congestion degree accurately. In addition, the backlog queue adjusts the congestion more smoothly and appropriately than traditional mechanisms that directly halve or even reduce *cwnd* to 1 to avoid congestion^31,32^. The reason is that the limited range of the backlog queue can prevent *cwnd* from infinitely growing until the true congestion occurs. In summary, the simulation results verify our previous assumption and thus motivate us to design TCP-WBQ using the backlog queue in (3) as the congestion recognition.

## TCP-WBQ mechanism

### Mechanism description

**Figure 3:**
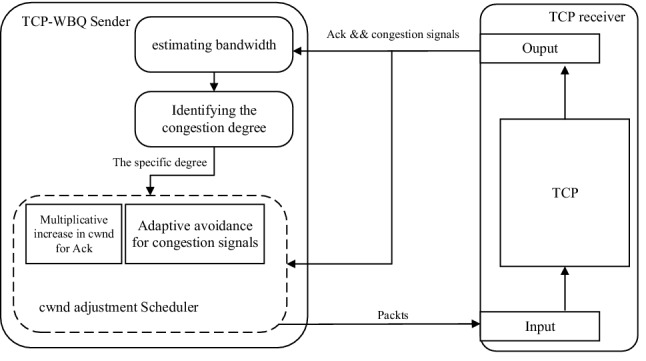
TCP-WBQ framework.

As aforementioned, the reason why TCP-W suffers from poor performance in heterogeneous wireless networks^33,34^ is that it depends upon the oscillated and unstable *RTT* or *EB* which are unable to reflect effectively the congestion degree. In order to solve the problem, we propose TCP-WBQ that inherits the three steps in “Motivation for TCP-WBQ” Section to detect congestion. The TCP-WBQ framework is as shown in Fig. 3.

TCP-WBQ adds the modular, i.e., identifying the congestion degree. While receiving normal Acks and congestion signals, TCP-WBQ estimates the backlog queue size (*q*) in (3) to identify the congestion degree. In addition, the periodicity of bandwidth estimations is each previous *Rtt*, not the interval of consecutive two Acks. The periodicity is the same as that in TCP-W + , which effectively resists *Rtt* fluctuations^35^ due to the random packet loss and high latency. Moreover, TCP-WBQ uses the *cwnd* adjustment scheduler to set *cwnd* and *ssthresh*. The scheduler implements different schemes for normal Acks and the congestion signals, respectively.

### Identifying congestion degree

#### Backlog queue bounds

In heterogeneous wireless networks, the unavoidable random packet loss on the wireless link, derived from random packet loss, can frequently trigger the unnecessary congestion avoidance and thus results in the backlog queue fluctuating in a limited range. In this section, we’ll discuss the backlog queue length only affected by the random packet loss. From (3), the maximum and minimum backlog queues are shown as

4 $$q_{\min } (t) = w_{\min } (t) - c_{act} (t)Rtt(t)$$

5 $$q_{\max } (t) = w_{\max } (t) - c_{act} (t)Rtt(t)$$

If the backlog queue reaches its maximum and minimum at the time *t*_*1*_ and *t*_*2*_ respectively, its limit thus will be

6 $$\begin{aligned} L & = q_{\max } (t_{1} ) - q_{\min } (t_{2} ) \\ & = w_{\max } (t_{1} ) - c_{act} (t_{1} )Rtt(t_{1} ) \\ & \quad - w_{\min } (t_{2} ) + c_{act} (t_{2} )Rtt(t_{2} ) \\ \end{aligned}$$

The backlog queue resizes within the limited range that is only determined by the random packet loss. Since true congestion does not occurs, we suppose that the number of outgoing packets per *Rtt* is equal and *Rtt*s are all the same. Therefore, (6) is written as

7 $$L = w_{\max } (t_{1} ) - w_{\min } (t_{2} )\quad s.t.\;w_{\min } \ge c_{act} (t)Rtt(t)$$

To determine the range *L*, we first define the decline ratio of *cwnd* due to the random packet loss. Since *cwnd* drops to 1/*β* of its size for each random packet loss, the 1/*β* is the decline ration. Each packet in *cwnd* has the probability *p* to be dropped randomly in wireless links, which means each packet follows a binomial distribution. Meanwhile, all packets in *cwnd* cannot be lost, i.e., communication interruption rather than congestion. Consequently, the expected decline ratio is as follows.

8 $$\begin{aligned} S & { = }\sum\limits_{{{\text{m}} = 1}}^{{w_{\max } - 1}} {\left( {\frac{1}{\beta }} \right)^{m} \left( \begin{gathered} w_{\max } - 1 \hfill \\ m - 1 \hfill \\ \end{gathered} \right)} (1 - p)^{{w_{\max } - m}} p^{m - 1} \\ & = \frac{1}{\beta }\sum\limits_{{{\text{m}} = 0}}^{{w_{\max } - 1}} {\left( {\frac{p}{\beta }} \right)^{m} \left( \begin{gathered} w_{\max } - 1 \hfill \\ m \hfill \\ \end{gathered} \right)} (1 - p)^{{w_{\max } - 1 - m}} \\ & = \frac{1}{\beta } \times \left[ {\frac{p}{\beta } + (1 - p)} \right]^{{w_{\max } - 1}} \\ & { = }\frac{{{[}\beta + (1 - \beta )p]^{{w_{\max } - 1}} }}{{\beta^{{w_{\max } }} }} \\ \end{aligned}$$

where *p* as the random-loss probability of each packet. In conventional AIMD mechanisms, e.g., TCP-NewReno, *β* is set to 2.

According to the definition in (6), *L* means a limited capacity for accommodating the backlog packets waiting for being sent during a certain time. Also because *c*_*act*_∙∆*t* is the number of transmitted packets during ∆*t* and *S* is the expected declined ratio due to random packet loss, the product of them means the number of randomly dropped packets during ∆*t*. These dropped packets cumulate a queue, i.e., the backlog queue, so the limited range of the backlog queue, *L*, equals the product of the decline ratio, *c*_*act*_, and the transmission time, as is shown

9 $$L = S \cdot c_{act} \cdot \Delta t$$

Substituting (8) into (9), we have

10 $$L{ = }\frac{{{[}\beta + (1 - \beta )p]^{{w_{\max } - 1}} }}{{\beta^{{w_{\max } }} }}c_{act} \Delta t$$

Let *r* represent one *Rtt*, and *k* is a positive integer. Since *∆t* ∈ [*r, kr*], the range of (10) is also denoted by the inequality

11 $$\frac{{{[}\beta + (1 - \beta )p]^{{w_{\max } - 1}} }}{{\beta^{{w_{\max } }} }}c_{act} r \le L \le \frac{{{[}\beta + (1 - \beta )p]^{{w_{\max } - 1}} }}{{\beta^{{w_{\max } }} }}c_{act} kr$$

The *cwnd* updates at every *Rtt* in TCP-WBQ, and grows to *w*_*max*_ spending at most *kr* time, i.e., *k *$\le$* w*_*max*_/*c*_*act*_*r*. As a result, the max limit of *L* is shown

12 $$L \le \frac{{{[}\beta + (1 - \beta )p]^{{w_{\max } - 1}} w_{\max } }}{{\beta^{{w_{\max } }} }}$$

-In (12), *p* is generally small, so

$$\frac{{{[}\beta + (1 - \beta )p]^{{w_{\max } - 1}} w_{\max } }}{{\beta^{{w_{\max } }} }} \approx \frac{{w_{\max }^{{}} }}{\beta }$$

_and thus_

13 $$L \le \frac{{w_{\max } }}{\beta }$$

Note that *L* is a range of the backlog queue evolving for the random packet loss. Alternatively, we obtain the max value of *cwnd*

14 $$\begin{aligned} & q_{\max } (t_{1} ) - q_{\min } (t_{2} ) \le \frac{{w_{\max } }}{\beta } \\ & \Rightarrow w_{\max } (t{ + }k \cdot Rtt) - w_{\min } (t) \le \frac{{w_{\max } }}{\beta } \\ & \Rightarrow w_{\max } (t{ + }k \cdot Rtt) \le c_{act} Rtt{ + }\frac{{w_{\max } }}{\beta } \\ & \Rightarrow w_{\max } \le c_{act} Rtt{ + }\frac{{w_{\max } }}{\beta } \\ & \Rightarrow w_{\max } \le \beta c_{act} Rtt/(\beta - 1) \\ \end{aligned}$$

Since *w*_*max*_ is the limited range of *cwnd*, TCP-WBQ uses its supremum to tolerate the *cwnd* fluctuations furthest. Thus, the upper bound of the backlog queue for random packet loss is

15 $$q_{\max } (t) = c_{act} (t)Rtt/(\beta - 1)$$

Because of the prevalence of AIMD, *β* is set 2 for TCP-WBQ to be friendly to other TCP variants. Thus, (14) is rewritten as *w*_*max*_(*t*) = 2*c*_*act*_(*t*)*Rtt*; (15) is rewritten as *q*_*max*_(*t*) = *c*_*act*_(*t*)*Rtt*.

According to (3), the backlog queue’s formation means the pack loss has occurred because the bandwidth is less than the transmission rate at the sender. The minimum bound of the backlog queue is that the backlog queue has just formed but its length is still zero, which is the threshold between non-congestion and packet loss. The threshold in terms of the backlog queue, *q*_*thresh*_, is defined as

16 $$q_{thresh} = 0\quad s.t.\quad w(t) = a_{act} (t)Rtt\left( t \right)$$

Accordingly, the value of *cwnd* in *q*_*thresh*_ is denoted as

17 $$w_{thresh} {(}t{) = }c_{act} (t)Rtt(t)$$

#### Identifying

In the previous section, we have deduced two respective bounds for random packet loss and non-congestion in the backlog queue, as well as the corresponding *cwnd*. Next, we will leverage the two bounds to identify three congestion degrees, as followed.

- *Non-congestion* the backlog queue unformed means congestion has not occurred because the available bandwidth is more than the transmission rate at the sender. i.e., *q*(*t*) $\le$ *q*_*thresh*_, or *w*(*t*) $\le$ *w*_*thresh*_.
- *Spurious congestion* the backlog queue size is between zero and *q*_max_, i.e., *q*_*thresh*_ < *q*(*t*) $\le$ *q*_max_, or *w*_*thresh*_ < *w*(*t*) $\le$ *w*_*max*_. Spurious congestion means that data streams are not congested although the backlog packets are formed because of random packet loss which traditional TCP mechanisms treat as a congestion event.
- *True congestion* the backlog queue is more than *q*_max_, i.e., *q*_max_ < *q*(*t*), or *w*_*max*_ < *w*(*t*). Because *q*_max_ is the max limit of the backlog queue oscillations due to the random packet loss, the excess size is derived from communications congestion.

Therefore, TCP-WBQ determines the specific congestion degree by the size of the backlog queue instead of unreliable and inaccurate congestion signals.

### Adjusting *cwnd*

In this section, we discuss how TCP-WBQ implements, for the full utilization of capacity, the multiplicative increase in *cwnd* rather than the additive increase of TCP-W in the case of non-congestion, and the adaptive congestion avoidance for different congestion signals.

#### Multiplicative increase

Typically, most popular TCP variants adopt the additive increase in *cwnd* when receiving each Ack of sent packets. To compete with these TCP variants on the shared bottleneck link in the heterogeneous network, TCP-WBQ uses the multiplicative increase to fully utilize capacity, which either greatly raises *cwnd* compared to the additive increase or tries the best to avoid inducing the true congestion.

Similar to TCP-W, TCP-WBQ still adjusts *cwnd* when every Acks arrives at the TCP sender. And TCP-WBQ constantly changes *cwnd* according to the congestion degree, i.e., the size of the backlog queue. In the cases of the spurious congestion and true congestion, keeping *cwnd* at *w*_*thresh*_, i.e., the backlog queue size is *q*_*thresh*_, is a tradeoff between high throughput and traffic congestion. On the contrary, since the backlog queue is still unformed in the case of non-congestion, directly increasing *cwnd* to *w*_*max*_ can rapidly increase throughput, which is beneficial to the wireless links with random packet loss. Since the random loss induces unnecessary congestion avoidance and therefore reduces throughput, the multiplication of increasing *cwnd* to *w*_*max*_ can ignore the effect of random packet loss to resist the *cwnd* fluctuations, and thus compensates for the throughput degradation. The multiplicative increase in *cwnd* is shown by (18) when the TCP sender receives each Ack.

18 $$w_{i} (t) = \left\{ {\begin{array}{*{20}c} {w_{\max } (t)} & {if\;q_{i} (t) = 0} \\ {w_{thresh} (t)} & {if\;q_{i} (t) > 0} \\ \end{array} } \right.$$

Specifically, the multiplicative increase in (18) uses the current *Rtt* rather than *Rtt*_min_ in TCP-W, the change that is adaptable to the different congestion degrees and therefore can effectively raise *cwnd*.

#### Adaptive Congestion avoidance

As to congestion avoidance, the prevalent mechanisms plunge *cwnd* down to avoid congestion when triple duplicate Acks arrive or RTO events occur. Since these mechanisms cannot discriminate the spurious congestion induced by the random packet loss, the direct cut of *cwnd* over-responds to the congestion signals, thereby frequently suppressing the transmission rate. To overcome the defect, TCP-WBQ implements two separate procedures to avoid congestion adaptively, based on the degree of congestion and the type of the congestion signals. In detail, that the procedure responding to triple duplicate Acks avoids congestion is exhibited by Algorithm 1. The second procedure avoiding congestion considers the RTO event. Same as the first, it also implements the adjustment in *cwnd* combining with the three congestion degrees. The second procedure, in true congestion, still implements the traditional *cwnd* decrease for RTO, that is, *cwnd* = 1, because the decrease effectively relieves congestion. However, in spurious congestion, *cwnd* only decreases by half. This action considers RTO is only derived from packet reordering due to asymmetry communications in heterogeneous networks. In the case of non-congestion, the second procedure performs the same multiplicative increase as that in  “Multiplicative increase” Section, because no congestion occurs. The specific implementation for RTO is shown in Algorithm 2. 
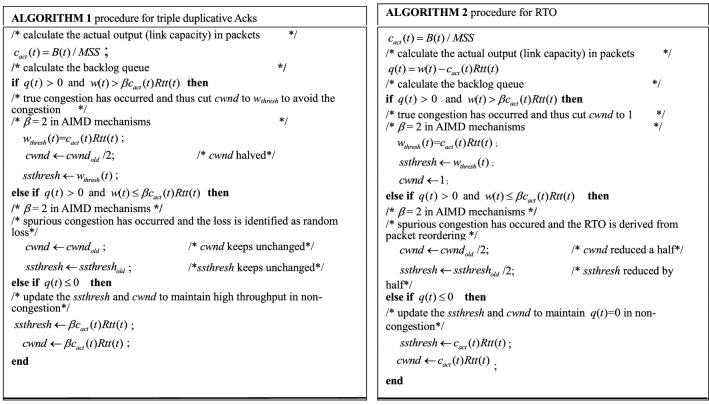


### Analysis of TCP-WBQ

In order to show the competitiveness of TCP-WBQ on the bottleneck link, we demonstrate TCP-WBQ’s benefit in terms of recovery time and the average throughput by mathematically analyzing the growth of *cwnd* in TCP-WBQ. The analysis method are based on the model in^34^. The model considers the *cwnd* variation is a stable system, and thus the average interval of the system encountering congestion is the same. The model includes two periods which transfer in circles. One is the recovery phase in which *cwnd* grows from the reduced value to the expected maximum. The reduced value is half of the expected maximum in AIMD. The other is the stable phase in which *cwnd* remains unchanged at the expected maximum. Additional, once the system encounter congestion, the stable phase transfers to the recovery phase. To conveniently analyze, we define the time during the recovery phase as $\Delta t_{1}$, and the time during the stable phase as $\Delta t_{{2}}$.

#### Analysis of recovery time

In TCP-WBQ, *w*_*thresh*_ is the reduced *cwnd* for the true congestion, and *w*_max_ is the expected maximum because it limits the max size of the backlog queue.

During *cwnd* growing from *w*_*thresh*_ to *w*_*max*_ in TCP-WBQ, the value of *cwnd, w*_*i*_(*t*), always keeps 2*c*_*act*_(*t*)*Rtt*(*t*) ($\beta = 2$) when every Ack of sent packets arrives. Therefore, *w*_*i*_(*t*) is formulated by

19 $$w_{i} (t) = \left\{ {\begin{array}{*{20}c} {\frac{{w_{thresh} (2Rtt)^{\frac{t}{Rtt}} }}{Rtt}} & {if\;w_{i} (t) \le w_{\max } } \\ {w_{\max } } & {otherwise} \\ \end{array} } \right.$$

In the recovery phase, since *w*_*thresh*_ = *c*_*act*_(*t*)*Rtt*(*t*) which is *w*_*max*_/2, the time $\Delta t_{1}$ in TCP-WBQ is

20 $$\frac{{w_{\max } }}{2Rtt}(2Rtt)^{\frac{t}{Rtt}} \left| \begin{gathered} \hfill \\_{t = Rtt} \hfill \\ \end{gathered} \right. = w_{\max }$$

Thus, the recovery time for *cwnd* approaching *w*_*max*_ is only one *Rtt*. However, in AIMD mechanism, *w*_*i*_(*t*) growing during one *Rtt* is

21 $$w_{i} (t) = \frac{{w_{\max } }}{2}{ + }1$$

From (21), the recovery time in AIMD is *w*_*max*_/2∙*RTT*. Because *w*_*max*_ is often much more than two packets, the recovery time in TCP-WBQ is much faster and is just one *Rtt* to recover to *w*_*max*_ rather than several *Rtts* as in AIMD mechanism. This is important to the TCP stream via the high-latency wireless link when it competes with other streams on superior wired links.

#### Analysis of average throughput

Supposing the probability of each packet suffering from the random loss is *p*, which meets (22) according to^34^.

22 $$\frac{1}{p} = s1 + s2$$

In (22), *s*1 and *s*2 are the numbers of transmitted packets in the recovery and stable phases. As to TCP-WBQ, the time of s1, $\Delta t_{1}$ , is equal to one *Rtt* according to (20). Thus, s1 is

23 $$\begin{aligned} s1 & = \int_{0}^{Rtt} {\frac{{w_{\max } }}{2Rtt}} (2Rtt)^{t/Rtt} dt \\ & = \frac{{w_{\max } }}{2\ln (2Rtt)}(2Rtt - 1) \\ \end{aligned}$$

In TCP-WBQ, since s2 = $\frac{{w_{\max } }}{Rtt}\Delta T_{2}$, the time $\Delta t_{{2}}$ can be obtained from (22) and (23)

24 $$\Delta T_{2} = \frac{Rtt}{{pw_{\max } }} + \frac{Rtt(1 - 2Rtt)}{{2\ln (2Rtt)}}$$

Therefore, the average throughput in TCP-WBQ is

$$\begin{aligned} Th^{TCP - WBQ} & = \frac{s1 + s2}{{(\Delta T_{1} + \Delta T_{2} )}} \\ & = \frac{1}{{\frac{Rtt}{{w_{\max } }} + Rtt \cdot p\left[ {1 + \frac{1 - 2Rtt}{{2\ln (2Rtt)}}} \right]}} \\ \end{aligned}$$

_Since *Rtt* and *p* are both very small, so_

25 $$Th^{TCP - WBQ} \approx \frac{{w_{\max } }}{Rtt}$$

(25) reveals that the average throughput of TCP-WBQ is approximately independent upon the probability of the random packet loss. This further explains the advantage of TCP-WBQ on wireless links.

#### Analysis of fairness

The average throughput of TCP-W is shown in^34^, which is

26 $$Th^{westwood} = \frac{{w_{\max } }}{{Rtt + \frac{{pw^{2}_{\max } }}{2Rtt}(1 + \frac{{Rtt_{\min }^{{}} }}{Rtt})^{{2}} }}$$

Since TCP-NewReno adopts AIMD mechanism, the mechanism directly reduces *cwnd* in half. So, in TCP-NewReno,$\Delta t_{1}$ is the time in which *cwnd* linearly grows from *w*_max_/2 to *w*_max_ other than from *w*_max_*Rtt*_*min*_/*Rtt* to *w*_max_ in TCP-W. Therefore, *Rtt*_*min*_ in (26) is equivalent to *Rtt*/2 in TCP-NewReno, and the average throughput of TCP-NewReno is

27 $$Th^{tcp - newreno} = \frac{{w_{\max } }}{{Rtt + \frac{{{9}pw^{2}_{\max } }}{{{8}Rtt}}}}$$

From (25–27), TCP-WBQ’s throughput is more than the other two TCP variants in the condition of the same *Rtt* and *w*_max_. The reason is its *cwnd* reaches *w*_max_ as fast as possible and is limited by 2*c*_*act*_(*t*)*Rtt*(*t*) to avoid true congestion. Additionally, if the probability of random packet loss (*p*) is small, the average throughputs of the three TCP variants approximate each other. These demonstrate that TCP-WBQ on wireless links maintains good fairness with other prevalent TCP variants.

## Evaluation

We present our simulation results and evaluate TCP-WBQ in two simulations that include the two typical topologies of heterogeneous wireless networks, i.e., the dumbbell and the parking lot. In the dumbbell topology including the single bottleneck, we compare various TCP variants according to different metrics, including the average throughput, the real-time estimated bandwidth, the normalized backlog queue, and the competitive fairness. The compared TCP variants include TCP-NewReno, Hybla, and TCP-W, which are widely applied to heterogeneous wireless networks. Meanwhile, we also compare Westwowd-ekf^36^ in simulations, which uses extended Kalman Filter instead of Tustin Filter in TCP-W to compensate for the random packet loss. In the other topology of the parking lot which means multiple bottlenecks, we compare the prevalent TCP variants in wireless and large bandwidth delay product (BDP) networks, which include TCP-BBR, TCP-Hit, and TCP-Veno. The simulation platform uses the Network Simulator with version 3.35 (NS3). In “Topology of single bottleneck and parameters” Section, we detail the single bottleneck’s topology and simulation parameters. Comprehensive simulations in terms of throughput are implemented for TCP variants with different communication delays and packet losses in “Performance with different traffic” Section. “Variation of the backlog queue” Secion reveals the evolution of the normalized backlog queue for all compared TCP variants in conditions of moderate and heavy traffic. The competitive fairness of TCP-WBQ and other variants over a bottleneck link is tested in “Competitive fairness” Section. At last, compared with other latest TCP variants in wireless BDP networks and datacenter networks in multiply bottlenecks, TCP-WBQ’s goodput has the obvious advantage.

### Topology of single bottleneck and parameters

**Figure 4:**
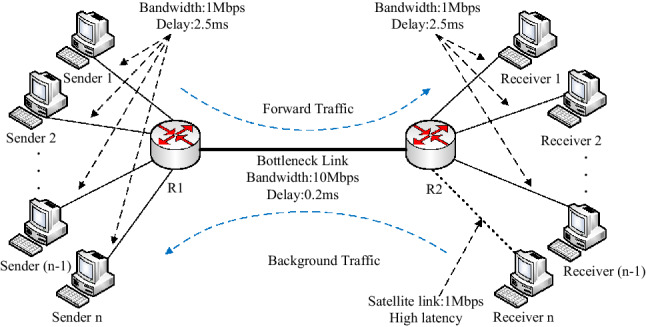
The dumbbell topology of simulation.

The dumbbell network is the most prevalent topology with a single bottleneck in wireless heterogeneous networks. It includes a bottleneck link on which streams compete with each other, easily inducing congestion. In our simulation topology shown in Fig. 4, *n* senders {n_1_, n_2_ …} and *n* receivers connect to each other by a bottleneck link which includes two routers {R_1_, R_2_}. The *n*th receiver connects to R_2_ by a wireless link with high delay and random packet loss. Note that the simulations have an opinion that each receiver also can send a UDP stream to the corresponding sender. These UDP streams form reverse background flows, which can compress the Acks and prevalently exists in real communications.

Unless mentioned otherwise, the following parameters are set as default: MSS is 536 bytes; the wired link’s latency is 2.5 ms, and its capacity is 1 Mbps; the bottleneck link capacity is 10 Mbps, and its delay is 0.2 ms; the probability of packet loss (*pl*) on the bottleneck link is 0.001%; *n* is set 10 and 20 to represent the moderate and heavy traffic, respectively.

### Performance with different traffic

In each test, a total of 180 runs, each lasting 200 s and using different seeds for generating *pl* on the wireless link, have been performed to compute the average throughput of each TCP variant mentioned above.

**Figure 5:**
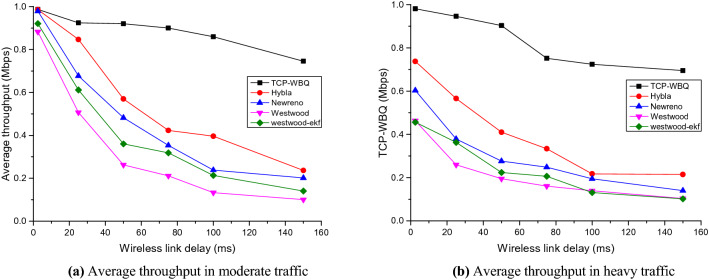
Average throughput of all compared TCP variants against wireless latency in different traffic for *pl* = 0%.

**Figure 6:**
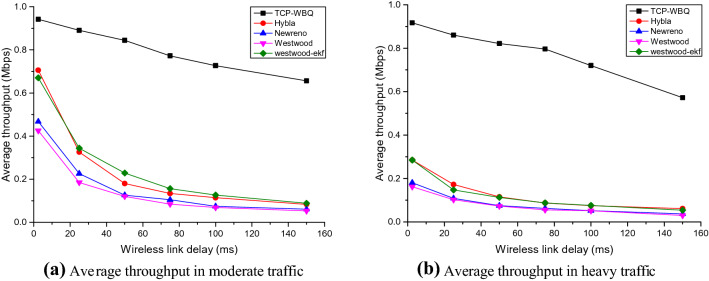
Average throughput of all compared TCP variants against wireless latency in different traffic for *pl* = 0.006%.

**Figure 7:**
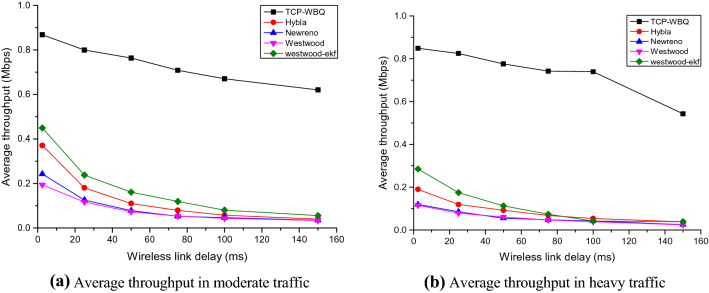
Average throughput of all compared TCP variants against wireless latency in different traffic for *pl* = 0.01%.

The performance test includes two scenarios, i.e., the moderate traffic (*n* = 10) without reverse background flows and the heavy traffic (*n* = 20) with reverse background flows. The wired TCP flows, controlled by TCP-NewReno, compete with the *n*th wireless TCP flow driven by other TCP variants. Figures 5, 6, and 7 plot the *n*th flow’s average throughput against the wireless latency in the two scenarios, for *pl* = 0%, 0.006%, and 0.01%.

In moderate and heavy traffic, all TCP variants degrade the performance to different extents as the wireless link’s delay increases. In Figs. 5, 6 and 7, TCP-WBQ, compared with other variants, demonstrates the highest throughput in all cases. The reason for outperformance is twofold. First, TCP-WBQ can distinguish and ignore the random packet loss in the case of the non-congestion. The second reason is that it takes one *Rtt* to recover *cwnd* from the half caused by the spurious congestion. As to other TCP variants, TCP-NewReno and TCP-W both indistinguishably respond to the random packet loss and thus frequently reduce *cwnd*, resulting in the low throughputs as delay and *pl* increase. Although Westwood-ekf adopts extended Kalman Filter instead of Tustin Filter to shield the impact of the random packet loss, it recovers *cwnd* to *w*_max_ spending several *Rtts* due to lack of the rapid recovery mechanism in TCP-WBQ. These reasons result in TCP-WBQ outperforming other TCP variants in terms of average throughput.

**Figure 8:**
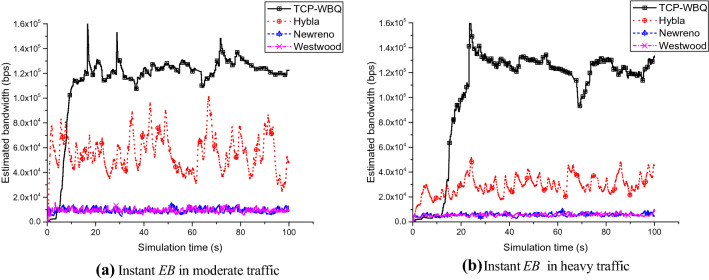
Instant *EB* of all compared TCP variants against time in different traffic for delay = 25 ms and *pl* = 0.004%.

**Figure 9:**
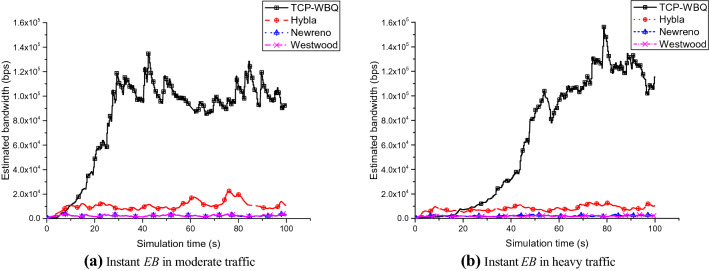
Instant *EB* of all compared TCP variants against simulation times in different traffic for delay = 100 ms and *pl* = 0.01%.

Figure 8 shows the instant *EB* in the TCP variants. TCP-WBQ features the highest bandwidth compared with others. As shown in Fig. 8a, in the case of moderate traffic, the *EB* of TCP-WBQ is almost twice that of Hybla. Similarly, the gap is four times in the heavy traffic, and TCP-WBQ in terms of bandwidth has more advantages compared with TCP-Newreno and Westwood, as shown in Fig. 8b. Figure 9 also exhibits the outperformance of TCP-WBQ over the wireless link for delay = 100 ms and *pl* = 0.01%. The bandwidth of TCP-WBQ increases rapidly and then keeps the value within a certain range. The results illustrate that TCP-WBQ best utilizes the capacity of the wireless link in these TCP variants. Conversely, other TCP variants mistakenly trigger congestion avoidance caused by the random packet loss so that their performances are inferior. And TCP-WBQ avoids the rapid decline of bandwidth because the backlog queue decides the congestion degree.

### Variation of the backlog queue

This simulation tests the variations of the backlog queue in different TCP variants, with the changes in the wireless link delay and packet loss for the two traffic scenarios.

We normalize the backlog queue to reveal the evolution of the backlog queue conveniently and accurately, as shown by

28 $$q^{normal} (t){ = }C\frac{q(t)}{{c_{act} (t)}}$$

*C* is the capacity of the link connecting the sender. Considering that the sizes of backlog queues cannot be directly compared due to different link capacities, the normalized backlog queue is proposed to represent the congestion level by the same criteria. Specially, we respectively implement two test sets for the size of the normal backlog queue in moderate and heavy traffic, with delay = 25 ms and *pl* = 0.002%, and delay = 100 ms and *pl* = 0.01%.

**Figure 10:**
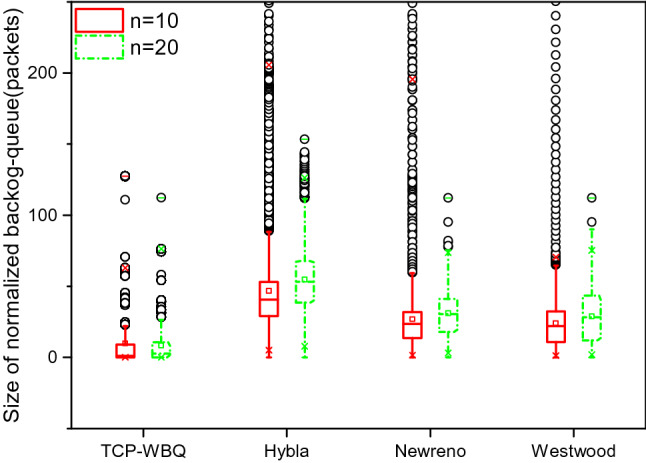
Variation of normalized backlog queue for all compared TCP variants.

**Figure 11:**
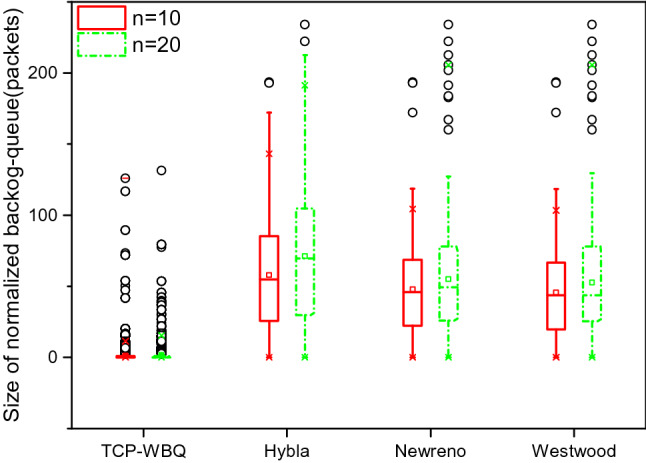
Variation of normalized backlog queue for all compared TCP variants in moderate and heavy traffic, with delay = 25 ms*, and pl* = 0.002% in moderate and heavy traffic, with delay = 100 ms, and *pl* = 0.01%.

In Fig. 10, the normalized backlog queue in TCP-WBQ is much smaller than those in other TCP variants. Furthermore, its fluctuation also maintains a small limited range. This is because TCP-WBQ adaptively adjusts *cwnd* to avoid congestion effectively. However, the normalized backlog queue in TCP-Hybla fluctuates severely, and its size is the largest because its *cwnd* grows rashly such that it frequently induces the true congestion. Both TCP-W and TCP-NewReno formed long backlog queues which represent high congestion levels. Figure 11 also demonstrates a similar result. The size of the normalized backlog queue in TCP-WBQ is steady and shortest, representing the lowest level of congestion.

### Competitive fairness

In the topology, the *n*th TCP stream competes against other *n-*1 streams for the bottleneck link’s capacity. Thereupon, we respectively examine the competitive fairness of different TCP variants by Jain’s fairness metric^37^, *J*, defined as

29 $$J(x_{1} ,x_{2} , \ldots ,x_{N} ) = \frac{{(\sum\nolimits_{i = 1}^{N} {x_{i} } )^{2} }}{{N \cdot \sum\nolimits_{i = 1}^{N} {x_{i}^{2} } }}$$

where *x*_*i*_ is the throughput of the *i*th TCP stream. And the *n*th TCP stream passing the wireless link respectively loads TCP-WBQ, TCP-NewReno, and Hybla, while other *n*−1 flows over wired links load TCP-NewReno. That *J* is close to 1 means the throughput of the *n*th TCP stream approximates that of other competitive streams. The higher value of *J* represents the higher competitive fairness.

**Figure 12:**
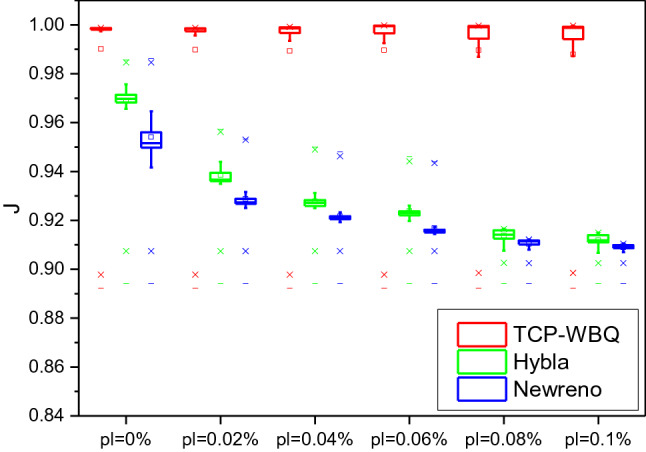
Jain’s fairness of compared TCP variants over the wireless link.

**Figure 13:**
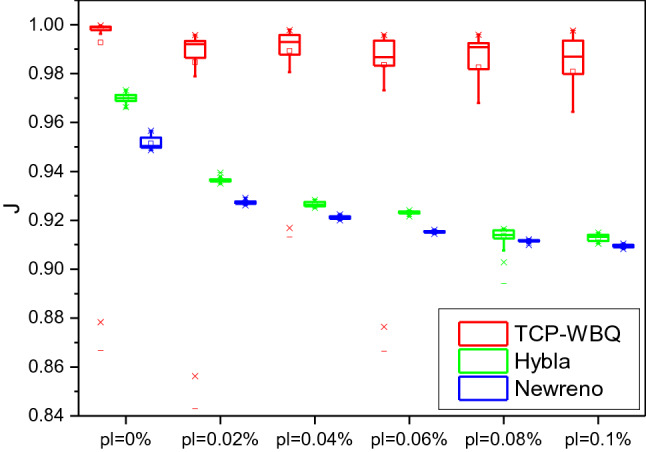
Jain’s fairness of compared TCP variants over the wireless link with delay = 25 ms and *n* = 20 with delay = 100 ms and *n* = 50.

Figure 12 plots the real-time *J* against *pl* over the wireless link with delay = 25 ms and *n* = 20. *J* of TCP-WBQ steadily approaches 1, and its fluctuation slightly grows as *pl* increases from 0 to 0.1%, meaning the minimal impact of *pl* on the TCP-WBQ stream. Compared with the fairness of TCP-WBQ, that of Hybla and TCP-NewReno drastically decreases. This means that the two variants underutilize the capacity of the bottleneck link. Figure 13 illustrates the fairness of compared TCP variants over the wireless link with delay = 100 ms and *n* = 50. In the same way, *J* of TCP-WBQ outperforms that of the other two TCP variants. The results demonstrate that TCP-WBQ over wireless links qualifies to fully compete with conventional TCP-NewReno or variants using AIMD over wired links.

So far, we can observe that TCP-WBQ attains significant performance gain, minimum backlog queue, and most competitive fairness over the wireless link with different latency and *pl*. These results prove TCP-WBQ effectively shields the impact of the random packet loss and high latency on the wireless link, so it is very suitable for heterogeneous wireless networks.

### Goodput in multiple bottlenecks

**Figure 14:**
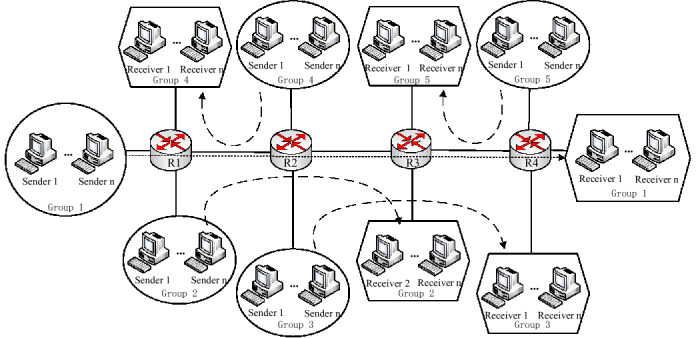
The parking lot topology of simulation.

We use a parking lot topology with multiple bottlenecks to reveal the satisfied goodput of TCP-WBQ in heterogeneous wireless networks. Since multiple bottlenecks epitomize the complex heterogeneous networks, we compared the latest TCP protocols (TCP-BBR^38^, TCP-Hit^39^, and TCP-Veno^40^) available on BDP networks, to which the wireless links belong, to reveal the generality of TCP-WBQ. The multiple bottlenecks topology is shown in Fig. 14. The simulation includes 5 flow groups, each of which includes n flows. The test flows in group 1 cross the most bottlenecks and load different TCP variants respectively. The other groups load TCP-NewReno, which is the same as the first simulation configuration. Each flow group generating traffic complies with passion distribution (λ = 8) to simulate the condition of heavy traffic.

According to the analysis in “Analysis of TCP-WBQ” Section, the average throughput or goodput of TCP-WBQ is approximately independent upon the random packet loss, which benefits the wireless traffic in heterogeneous networks. This further explains the advantage of TCP-WBQ on wireless links. To verify the conclusion, we change the probability of random packet loss and latency on bottleneck links to reveal the effect of the two negative factors of wireless networks on the goodput of TCP-WBQ. According to variations of the two factors, the test established three communication scenarios, i.e., normal communication (the first scenario), worse communication (the second scenario), and worst communication (the third scenario). In addition, n is set to 10 in each flow group and the simulation time is 100 s. We only select a performance-average stream in group 1 when the group loaded different TCP variants respectively.

**Figure 15:**
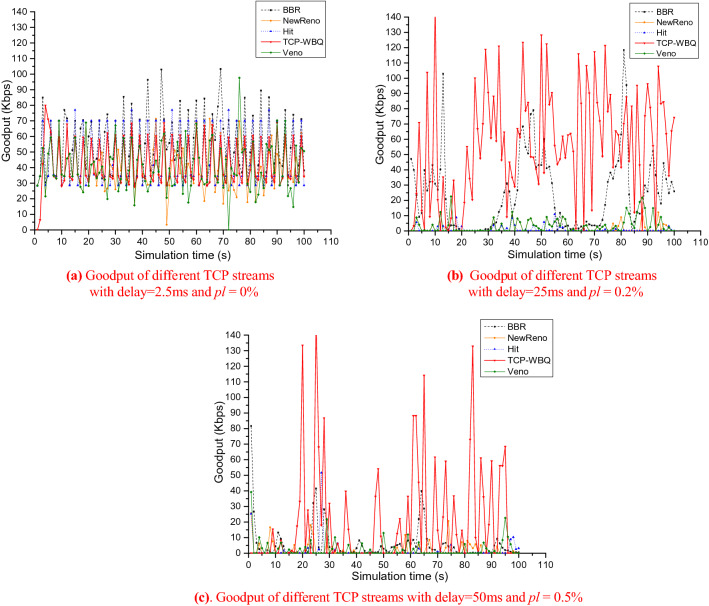
Goodput of all compared TCP variants over multiple bottleneck links.

The experiment results of the first scenario are shown in Fig. 15a. The results show that TCP-WBQ is comparable to TCP-NewReno, but TCP-BBR is much better than other TCP variants under the good communication condition. This is because TCP-BBR periodically estimates the available bandwidth and minimal round-trip time^41^. The good communication condition ensures the bottleneck remains saturated but not congested. Therefore, TCP-BBR can operate at Kleinrock’s optimal operating point in maximum throughput with minimal delay^42^. Meanwhile, the performances of other TCP variants have no significant difference.

As shown in Fig. 15b, the bottleneck links’ delay increases from 2.5 to 25 ms when *pl* becomes 0.2%, the poor condition that conforms to the wireless links in heterogeneous networks. Since the transmission quality of bottleneck links degrades so that other TCP variants’ goodput deteriorates to different degrees, TCP-WBQ implements the multiple *cwnd* increase and adaptive congestion avoidance to efficiently utilize the shared bandwidth of bottleneck links. The results verify that TCP-WBQ improves the TCP stream competitiveness in heterogeneous wireless networks.

In the third scenario, the bottleneck links’ delay and *pl* are separately changed to 50 ms and 0.5% to simulate a serious wireless communication full of competition. The experiment results are shown in Fig. 15c. Although all TCP variants have degraded performances due to the worst latency and random packet loss, TCP-WBQ’s goodput significantly prevails over that of the other variants. The figure coheres with the conclusion in “Analysis of TCP-WBQ” Section that when random packet loss increases, TCP-WBQ is approximately independent of its effect. The goodput of TCP-WBQ basically keeps the same as that under the good communication condition. This confirms the theoretical analysis that TCP-WBQ better resists random packet loss.

**Figure 16:**
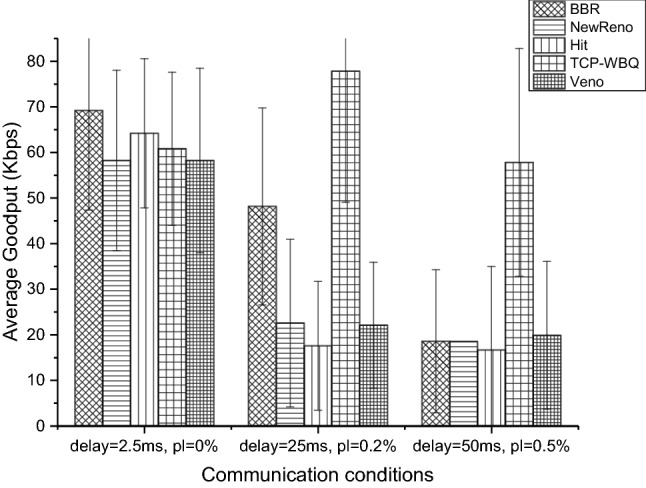
Average goodput of compared TCP variants in the multiple bottleneck scenarios.

The above experiment analyses are based on the performance-average stream in group 1. To better demonstrate the advantage of TCP-WBQ, we count the average goodput of all compared TCP variants in different scenarios. The statistical results are shown in Fig. 16. In the good communication scenario, the average goodput of all TCP variants is almost similar, where TCP-BBR performs better. With the communication condition deteriorating, TCP-WBQ remains satisfied goodput rather than significantly degrades performance like the other variants. The reason is that (1) TCP-WBQ effectively identifies the congestion degrees and then adaptively avoids congestion, which resists the effect of random packet loss. (2) With latency and packet loss of bottleneck links increasing, the competing TCP flows do not fully utilize the shared bandwidth, which results in TCP-WBQ can occupy more bandwidth resources by rapidly multiple increasing *cwnd*.

## Conclusion

In this paper, we have proposed a backlog-queue model at the TCP sender. Through simulation, we find that the size of the backlog queue is positively correlated with the congestion degree. Therefore, we propose a novel TCP variant (TCP-WBQ), which judges the congestion degree based on the backlog queue size, instead of the unreliable congestion signals, for congestion control in the heterogeneous wireless network. According to the congestion degree, TCP-WBQ multiplicatively increases *cwnd* to utilize the capacity fully, and implements the adaptive congestion avoidance for the different congestion signals. Our simulation investigation reveals that TCP-WBQ, in heterogeneous wireless networks, achieves significant performance improvement on the wireless link with the random packet loss and high latency. It also demonstrates TCP-WBQ’s good fairness in competing communications.
